# Supplementary material for: Short birth spacing and its association with maternal educational status, contraceptive use, and duration of breastfeeding in Ethiopia. A systematic review and meta-analysis
Source: PLoS One. 2021 Feb 3;16(2):e0246348. doi: 10.1371/journal.pone.0246348 (PMC7857626; doi:10.1371/journal.pone.0246348)
Supplement: S1 File — (DOCX) [file pone.0246348.s001.docx]

PubMed

(((((((proportion AND (english[Filter])) OR (prevalence AND (english[Filter]))) OR (magnitude AND (english[Filter]))) OR (incidence AND (english[Filter])) AND (english[Filter])) AND (((((((("Birth spacing" AND (english[Filter])) OR ("child spacing" AND (english[Filter]))) OR ("birth interval" AND (english[Filter]))) OR ("suboptimal birth intervals" AND (english[Filter]))) OR ("short birth spacing" AND (english[Filter]))) OR ("suboptimal child spacing" AND (english[Filter]))) OR ("optimal birth spacing" AND (english[Filter]))) OR ("inter-birth interval" AND (english[Filter])) AND (english[Filter]))) AND ((((("risk factors" AND (english[Filter])) OR (predictors AND (english[Filter]))) OR (factors AND (english[Filter]))) OR (determinants AND (english[Filter]))) OR ("associated factors" AND (english[Filter])) AND (english[Filter]))) AND ((("married women" AND (english[Filter])) OR (women AND (english[Filter]))) OR ("women of childbearing age" AND (english[Filter])) AND (english[Filter]))) AND (Ethiopia AND (english[Filter])) Filters applied: *Humans, Female, from 1990/1/1 - 2020/5/30*.

HINARI

((prevalence) OR (magnitude OR proportion) OR (incidence)) AND (("Birth spacing") OR ("child spacing") OR ("birth interval") OR ("suboptimal birth intervals") OR ("short birth spacing") OR ("suboptimal child spacing") OR ("optimal birth spacing") OR ("inter-birth interval)) AND (("risk factors") OR (predictors) OR (factors) OR (determinants) OR ("associated factors")) AND (("married women") OR (women) OR ("women of childbearing age")) AND (Ethiopian) Filters applied: *Humans, Female, from 1990/1/1 - 2020/5/30*.

Cochrane Library

**prevalence OR magnitude OR proportion OR incidence in All Text AND "Birth spacing" OR "child spacing" OR "birth interval" OR "suboptimal birth intervals" OR "short birth spacing" OR "suboptimal child spacing" OR "optimal birth spacing" OR "inter-birth interval" in All Text AND "risk factors" OR predictors OR factors OR determinants OR "associated factors" in All Text AND "married women" OR women OR "women of childbearing age" in All Text AND Ethiopian in All Text - with Cochrane Library publication date Between Jan 1990 and May 2020 (Word variations have been searched)** Filters applied: *Humans, Female, from 1990/1/1 - 2020/5/30*.
