# Supplementary material for: Short birth spacing and its association with maternal educational status, contraceptive use, and duration of breastfeeding in Ethiopia. A systematic review and meta-analysis
Source: PLoS One. 2021 Feb 3;16(2):e0246348. doi: 10.1371/journal.pone.0246348 (PMC7857626; doi:10.1371/journal.pone.0246348)
Supplement: Attachment — Submitted filename: Response to Reviewers.docx [file pone.0246348.s004.docx]

**Mohammad Rifat Haider (MBBS, MHE, MPS, PhD)**

**Academic Editor, PLOS ONE
RE: Manuscript ID: PONE-D-20-19678 (Short birth spacing and its association with maternal educational status, contraceptive use, and duration of breastfeeding in Ethiopia. A systematic review and meta-analysis).**

**Dear Dr. Mohammad,**

Thank you very much for your email and the comments/suggestions of the reviewers and academic editor. We have looked at the comments and have revised our paper accordingly. We hope our paper improved as a result of incorporating reviewer’s and academic editor’s comments and suggestions.

Please find for your kind consideration the following:

- A rebuttal letter that responds to each point raised by the academic editor and reviewer. The point by points responses are written in *italic* font style.
- A revised manuscript with track changes.
- A revised paper without tracked changes
- The point by points responses are written in *italic* font style.

While hoping that these changes would meet with your favourable consideration, we are happy to hear if there are more comments and suggestions. Please do not hesitate to let us know if you have any questions.

Yours Sincerely,

Yitayish Damtie

School of Public Health, Wollo University

Dessie, Ethiopia

Tel:+251943517982

E-mail: [yitutile@gmail.com](mailto:yitutile@gmail.com)

**Point by point response**
**Additional Editor Comments**

Take care of the reviewers' comments. Put special emphasis on the English language and grammar.

- *We have tried our best to improve it accordingly.*

Journal Requirements:

When submitting your revision, we need you to address these additional requirements.

1. Please ensure that your manuscript meets PLOS ONE's style requirements, including those for file naming. The PLOS ONE style templates can be found at

<https://journals.plos.org/plosone/s/file?id=wjVg/PLOSOne_formatting_sample_main_body.pdf> and

<https://journals.plos.org/plosone/s/file?id=ba62/PLOSOne_formatting_sample_title_authors_affiliations.pdf>

- *We have tried to revise our manuscript according to PLOSONE formatting style.*

2. Thank you for including the statement that "The database search was conducted on 'The searching of research articles was carried out from April 1 up to May 30, 2020, and articles published until May 30, 2020, were included in the review.' Please revise this statement to clarify whether all databases were searched from inception, or if there were any limits placed on the publication dates in your search.

- *Thank you for remembering an important issue. As it is stated above, database search was carried out from April 1 up to May 30, 2020, and articles published from the 1990 to May 30, 2020 were included in the review but we have missed to write it in the manuscript.*

3. At this time, we ask that you please provide the full search strategy and search terms for at least one database used as Supplementary Information.

- *We have tried to attach the full search strategy as supplementary information.*

4. Please provide the results of the assessment of publication bias conducted in the meta-analyses. Please provide the funnel plots of the Egger's regression test as a separate Figure in your manuscript.

- *We have taken in to consideration and provide the funnel plots as a separate Figure in our*

*Manuscript.*

**Reviewer Comments to the Author**

**Reviewer#1:** 
A significant limitation of the manuscript in its current form is the English. There are many mistakes and inconsistencies that require an urgent revision by a professional copyeditor. The mistakes make the manuscript challenging to read and understand.

- *We have tried to improve it accordingly.*

**Abstract**
methods: “and determinants of birth spacing in Ethiopia...” is not true. The researchers pre-specified the factors they explored. Please clarify.

- *We acknowledged the problem and tried to correct it accordingly in the abstract.*

Results: please describe the number of articles included in the review and in the meta-analyses.

- *We have tried to describe it accordingly in the main document.*

Conclusion: "The practice of short birth spacing in Ethiopia was higher." Please elaborate on this phrase.

- *We have taken in to consideration and tried to improve it accordingly.*

**Introduction**

Given the considerable number of factors associated with short birth spacing, it is not clear why the authors chose to explore only maternal education, contraceptive use, and duration of breastfeeding. Please clarify what criteria were used to choose the aforementioned factors.

- *Thank you for your comment. As we have tried to justify it in the introduction, a wide range of maternal and service-related factors that affect short birth spacing were identified from the included studies. But, we only examine the association between short birth spacing and maternal education, contraceptive use, and duration of breast feeding since they were the most frequently mentioned factors and have controversial finding among the included studies. In addition to this, these three factors are clinically significant factors affecting short birth spacing. As a result, we have interested to test their statistical significance and settle the controversial finding they have across the included articles.*

**Methods**

Line 95: “Grey literature was identified through the input of content experts.” It is not clear who these experts were and how they were selected and/or approached. Moreover, it is not clear how many studies the experts included and what criteria was used to select the studies.

- *Thank you for your comment. Only one study with a case-control study design reporting determinants of birth spacing among women of reproductive age was identified from the input of the two content experts who are currently working in our organization and specialized in different areas of maternal and child health issues. They were selected based on their area of specialization and approached after explaining the objective of our study. The pre-set inclusion criteria’s were used to select the grey literature.*

Lines 99 to 105: the authors should include the exact search they used in the different databases. A list of terms is not enough. Moreover, please consider uploading the search as an additional file as this will save space.

- *We have tried to upload the exact search as an additional file accordingly.*

Line 105: “This systematic review and meta-analysis followed the Preferred Reporting Items for Systematic Reviews and Meta-Analysis (PRISMA) checklist.” The PRISMA checklist is preferred for systematic reviews and meta-analysis of experimental studies. The MOOSE guideline is preferred for Meta-Analysis of Observational Studies in Epidemiology (doi:10.1001/jama.283.15.2008). Please refer to the MOOSE reporting guidelines and use them to report the findings of your article.

- *The comment is accepted and addressed accordingly.*

Inclusion criteria: the review is supposed to be focused only on studies exploring maternal education, contraceptive use, and duration of breastfeeding. However, this is not reflected in the inclusion criteria. Please clarify.

- *Yes, definitely. The review focuses only on studies exploring maternal education, contraceptive use, and duration of breastfeeding. However, explaining it as an inclusion criteria would exclude many articles exploring only the prevalence of short birth spacing but not the factors that we have mentioned above.*

Study setting: I suggest the authors to move this information to the introduction.

- *The comment is accepted and addressed accordingly.*

Outcome measurement: There will be many different definitions or cut-off points among the three factors that the authors wanted to explore in their systematic review. For example, the authors will find studies exploring duration of breastfeeding with cut-off points of 6, 8, 12, 18, and 24 months. It is not clear why the authors pre-specified their cut-off point to be 24 months. Moreover, it is not clear why the authors pre-specified their education contrast to be formal education vs no formal education, or contraceptive use yes vs no. Pre-specifying these contrasts would exclude many articles. This should be explained in the limitations section. Additionally, this information is not explained in the inclusion criteria. Please clarify.

- *Yes, different cut-off points were used to define or categorize the three factors. But in our case, the cut-off points are not pre specified from the inception. Rather they are determined to be 24 months for the duration of breastfeeding, formal vs no formal education for maternal educational status and contraceptive use vs not use for contraceptive utilization after the review process since the majority of the included articles used these cut-off points to define the these factors.* *There is no need to explain the cut of points as an inclusion criteria as it exclude many articles.*

**Results**
The authors reported high heterogeneity among the included studies. Why did the authors decide to conduct meta-analysis instead of providing a narrative synthesis of the results? Please clarify this sensitive issue.

- *Thank you for raising an important issue. Yes it is undeniable that sever heterogeneity was reported among the included studies*. *The reason behind* *conducting meta-analysis instead of providing a narrative synthesis of the results in the presence of high heterogeneity is to achieve the ultimate aim of our study since the objective of our study is to determine the pooled prevalence of short birth spacing and its association with contraceptive use, educational status and duration of breastfeeding among reproductive age women. But, efforts like using random effect model, conducting subgroup analysis and meta-regression were considered to minimize the variation and to identify the source of heterogeneity among the included studies.*

From the first paragraph of Results, it seems that the authors conducted a two-step screening phase. First, they screened the titles, and second, they screened the abstracts of the publications. In systematic reviews, however, titles and abstracts are evaluated together. I believe that screening the references in the way the authors did could introduce major biases in the review results. Please explain the rationale behind the two-step screening process.

- *In the actual screening process, the title and the abstract of the articles were screened and evaluated together. You can confirm this by observing the PRISMA flow diagram (Fig1). But, to be specific, the number of articles excluded by the title and the abstract were narrated separately in the result section of the main document. We acknowledge the problem since it introduce wrong understanding for the readers and tried to revise it accordingly.*

First paragraph of Results: the authors over explain the flow diagram of the articles screened/included. This information could be easily summarized in Figure 1.

- *We have tried to minimize the narration accordingly.*

In lines 181 to 184, the authors say that they included 7 studies in the meta-analysis. It is not clear why the authors included only these 7 studies from the original 9 included studies. More concerning, in lines 189 and 190 the authors mention that they included 6 articles, not 7. This issue needs clarification.

- *No, we have not said that we included 7 studies in the meta-analysis. Rather we said, a total of 9 studies (Shallo and Gobena, Ejigu et al, Tsegaye et al, Ayane et al, Gebrehiwot et al, Yohannes et al, Hailu et al, Begna et al and Tessema et al) were included in this meta-analysis. Among these studies, 6 of them (Shallo and Gobena, Ejigu et al, Tsegaye et al, Ayane et al, Gebrehiwot et al and Yohannes et al) were included to estimate the pooled prevalence of short birth spacing and 6 among the 9 studies (Tsegaye et al, Ayane et al, Yohannes et al, Hailu et al, Begna et al and Tessema et al) were included to exam the association between maternal education, contraceptive use, and duration of breast feeding and short birth spacing.*

The authors included only three studies in the meta-analysis related to breastfeeding. In the limitations, please explain the limitations of conducting meta-analysis with low numbers of studies.

- *We acknowledge the problem and tried to explain the limitation in the main document.*

**Discussion**

Lines 228 & 229, and 247 & 248: there is no need to repeat the objective of the study.

- *We have tried to correct it accordingly in the main document.*

Line 241: “a subgroup analysis was done by residence (both urban and rural versus rural)”. It is not clear to me why the authors decided to explore this contrast. Why not to simply explore urban vs rural? Please clarify.

- *Thank you for your comment. This is based on the setting in which the studies were conducted. As we have stated it in the main document, six studies were included to estimate the pooled prevalence of short birth spacing. Among these studies, four of them considered both rural and urban women, two studies considered only rural women and no study considered only urban women. Due to this reason, subgroup analysis by residence was explored as both urban and rural vs rural.*

Lines 249 to 251 and 265 to 258: there is no need to repeat the results of the study.

- *We have tried to avoid the repetition accordingly*

**Conclusion**

“Based on the finding, the Ethiopian government should improve the existing family planning program for all women of childbearing age.” Given the considerable limitations of this meta-analysis, I believe the authors are overstating their conclusion. Please adjust in light of the limitations of the study.

- *We have tried to adjust it accordingly*.

**Reviewer#2:** 
**ABSTRACT:**

**Objectives:** This is ok.

- Thank you

**Methodology**: The authors should include the key words used in the search, should state the selection criteria, outcome measures assessed, the subjects of the studies they included in the meta-analysis and which outcomes were analyzed.

- *Yes, all the things that you have mentioned above are very important and must be included in the methodology part. However, PLOSONE formatting style (300 words in the abstract section) limit us to incorporate all the aforementioned comments under the abstract section. As much as possible, we have tried our best to include some of them.*

**Main results:** The authors should state the number of studies assessed, identified and how many were included in the meta-analysis.

- *We have tried to address it accordingly.*

What were the results of heterogeneity across studies?

- *We have tried to include the result of heterogeneity as per your comment.*

**CONCLUSION**: why did the authors state that: “the practice of short birth spacing in Ethiopia was higher.’ The authors should rephrase their conclusion because the practice of short birth spacing in Ethiopia cannot he said to be higher. The authors should avoid 'slangs' in their writing like ‘didn’t’.

- *We acknowledge the problem and we have taken in to consideration.*

**INTRODUCTION**
although, this is well written; however, the introduction needs brushing up of language and beefing up the justification for the study.

- *We have tried to improve it accordingly.*

**MATERIALS AND METHODS**

Why did the authors exclude relevant articles with full texts of which unavailable after two email contacts of the corresponding authors?

- *We exclude articles with full texts of which unavailable after two email contacts of the corresponding authors because we couldn’t find relevant variables on the abstract section to achieve the prime objective of our study like sample size and the number of women with short birth spacing.*

**RESULTS**
In table 1, why was the prevalence of the last three publications not included?

- *Yes, definitely. The prevalence of the last three publications was not included in the table. Because, prevalence of short birth spacing could not be calculated the in the case of studies with a case-control and retrospective follow up study design. But, we include these three studies to examine the association between short birth spacing with maternal educational status, contraceptive use, and duration of breastfeeding.*

**DISCUSSION**

The authors must correct the type errors and other errors in the manuscript. The authors need English language editor.

- *We have tried to improve the type and grammatical errors accordingly.*

The authors should discuss the clinical implications of their study findings.

- *We have accepted the comment and tried to discuss the clinical implication of our finding in the discussion section of the main document.*

The authors should also discuss the strengths of their study.

- *Thank you for your comment. Discussing the strengths of this study is not as such important since our study shared all the strengths of the systematic review and meta-analysis.*
