# Supplementary material for: Short birth spacing and its association with maternal educational status, contraceptive use, and duration of breastfeeding in Ethiopia. A systematic review and meta-analysis
Source: PLoS One. 2021 Feb 3;16(2):e0246348. doi: 10.1371/journal.pone.0246348 (PMC7857626; doi:10.1371/journal.pone.0246348)
Supplement: Attachment — Submitted filename: Response to Reviewers.docx [file pone.0246348.s005.docx]

**Mohammad Rifat Haider (MBBS, MHE, MPS, PhD)**

**Academic Editor, PLOS ONE
RE-2: Manuscript ID: PONE-D-20-19678R1 (Short birth spacing and its association with maternal educational status, contraceptive use, and duration of breastfeeding in Ethiopia. A systematic review and meta-analysis).**

**Dear Dr. Mohammad,**

Thank you very much for your email and the comments/suggestions of the reviewers and academic editor. We have looked at the comments and have revised our paper accordingly. We hope our paper improved as a result of incorporating reviewer’s and academic editor’s comments and suggestions.

Please find for your kind consideration the following:

- A rebuttal letter that responds to each point raised by the academic editor and reviewer(s) (labeled as Response to Reviewers).
- A marked-up copy of your manuscript that highlights changes made to the original version (labeled as Revised Manuscript with Track Changes).
- An unmarked version of your revised paper without tracked changes (labeled as Manuscript).
- The point by points responses are written in *italic* font style.

While hoping that these changes would meet with your favourable consideration, we are happy to hear if there are more comments and suggestions. Please do not hesitate to let us know if you have any questions.

Yours Sincerely,

Yitayish Damtie

School of Public Health, Wollo University

Dessie, Ethiopia

Tel:+251943517982

E-mail: [yitutile@gmail.com](mailto:yitutile@gmail.com)

**Point by point response**

**Additional Editor Comments**
Please take care of the reviewer's comment.

- *Thank you for your comment and suggestion, we have tried to incorporate all the reviewers comment in our revised manuscript.*

**Reviewer’s comments**

**Reviewer #1:**

The authors have addressed all my comments. There are still a few English mistakes. For example, "Short birth spacing had been linked" (line 63) -> has been linked. "Data source and searching strategies" (line 107) -> search strategy. I suggest the authors use a professional editing service to fine tune the manuscript.

- *Thank you for your constructive comments. We acknowledge the problem and we have tried to change “Short birth spacing had been linked" to has been linked and Data source and searching strategies" to search strategy on line number 66 and 102 of our revised manuscript respectively.*
- *We have tried our best to address the English mistakes in our manuscript*

**Reviewer #2:**

The authors have addressed the issues adequately. This is a systematic review aimed at estimating the pooled prevalence of short birth spacing and its association with contraceptive use educational status and duration of breastfeeding among reproductive-age women in Ethiopia.

Title: This is ok

- *Thank you in advance.*

Regarding the English language, the authors have endeavored to work on it.

- *Thank you for your comment. Efforts were made to address all the English mistakes and grammatical errors in our revised manuscript.*
